# *Effect* of *Capsicum annuum* on antioxidant activity, oocyte development and resistance against *Aeromonas hydrophila* in jewel cichlid (*Hemichromis guttatus*)

**DOI:** 10.1038/s41598-025-13481-7

**Published:** 2025-07-31

**Authors:** Nalan Ozgur Yigit, Secil Metin, Behire Isıl Didinen, Ozlem Ozmen, Rahime Aslankoc

**Affiliations:** 1 Egirdir Fisheries Faculty, Isparta University of Applied Sciences, Isparta, Turkey; 2 Department of Pathology, Faculty of Veterinary Medicine, Burdur Mehmet Akif Ersoy University, Burdur, Turkey; 3 Department of Physiology, Faculty of Medicine, Suleyman Demirel University, Isparta, Turkey

**Keywords:** Red pepper, Mature oocyte, Antioxidant, Jewel cichlid, Protection, Animal physiology, Ichthyology

## Abstract

This study aimed to determine the effects of 3%, 7%, 11%, and 15% red pepper (*Capsicum annuum*) supplementation to diet on antioxidant, oocyte development and disease resistance against *Aeromonas hydrophila* A210 of jewel cichlid (*Hemichromis guttatus*). Fifteen fish (3.43 g ± 0.03) were stocked to each aquarium and fed ad libitum for 90 days. At the end of the trial, the number of mature oocytes increased by adding 11% and 15% red pepper to the diets. (*P* < 0.05). Fish fed diets containing 11% and 15% red pepper showed a significant rise in superoxide dismutase (SOD) activity (*P* < 0.05). In addition, fish fed diets containing 3% and 11% red pepper showed disease resistance against *A. hydrophila* in jewel cichlids. After challenge with *A. hydrophila*, cumulative mortality rates in fish fed with diets containing 3%, 7%, and 11% red pepper were significantly reduced compared to the control group (*P* < 0.05). Therefore, using 11% red pepper is appropriate to provide resistance against *A. hydrophila* infections, increase fertility and antioxidant levels in jewel cichlids *(Hemichromis guttatus*).

## Introduction

Aquarium fish culture is among the fastest growing sectors in the world. Diseases in ornamental fish farming cause serious economic losses for fish breeders and hobbyists in many countries^1–3^. The most common diseases of ornamental fish are bacterial infections caused by *Aeromonas* species^3–5^. *Aeromonas hydrophila* is the predominant cause of Motile Aeromonas Septicemia (MAS). *A. hydrophila* is an opportunistic pathogen that could be easily be found in waters with high organic content. Stress and poor water quality significantly contribute to the development of MAS^5–8^. In recent years, the use of herbal medicines has been investigated as an alternative method for preventing fish diseases.

Red pepper is a plant or spice rich in compounds including carotenoids (β-carotene, lycopene, and astaxanthin), flavonoids (quercetin and luteolin), polyphenols, myricetin, vitamins C and E, provitamin A^9–11^. Carotenoids, one of the main active compounds of red pepper are natural immune stimulants that can improve antioxidant capacity and immune function^12–15^. In addition, red pepper is rich in phenolic compounds, including flavonoids and phenolic acids, which are linked to the prevention of various diseases^16^. Dietary supplementation of red pepper powder in zebrafish (*Danio rerio*) feed led to the improvement of immunity^17^. The use of red pepper extract is recommended to enhance the immune system of rainbow trout (*Oncorhynchus mykiss*)^15^.

Carotenoids also play a significant role in fish reproduction. According to research, adding carotenoids to fish diets enhances the reproduction of the fish^18,19^. Watanabe and Vassallo Agius^20^ reported that paprika powder supplementation improved egg production and egg quality of yellowtail (*Seriola quinqueradiata*). Scabini et al.^21^ also informed that carotenoids supplementation in diet improved reproductive performance in gilthead seabream (*Sparus aurata*). Fish cannot naturally synthesize carotenoids and therefore must obtain them from diet^22,23^.

Jewel cichlid (*Hemichromis guttatus*) is one of the fish species with high market demand among ornamental fish. The colors of jewel fish are very beautiful. It is a species native to West Africa. found in various streams, rivers and ponds in brackish waters^24,25^.

In previous studies, effects of growth and coloration of red pepper were investigated in blue streak hap *Labidochromis caeruleus*^26^, mozambiques tilapia,*Oreochromis mossambicus*^27^ and yellow tail cichlid, *Pseudotropheus acei*^28^. However, there are few studies about the effect of red pepper on disease resistance against *Yersinia ruckeri* in *Oncorhynchus mykiss*^15^ and *Aeromonas sobria* in *Pseudotropheus acei*^29^. Additionally, the effect of red pepper on reproductive performance has only been investigated in *Seriola quinqueradiata*^20,30^. In this study, the effects of supplementing red pepper on antioxidants, oocyte development, and disease resistance against *A. hydrophila* in jewel cichlids (*Hemichromis guttatus*) are investigated for the first time.

## Materials and methods

### Trials diets

Five isonitrogenic (370 g kg^− 1^) and isoenergetic (3831 kcal kg^− 1^) diets were formulated with different levels (0, 3, 7, 11 and 15%) of red pepper. Approximate composition of diets is shown in Table 1. Red pepper used in the study was obtained from a local market. The fresh red peppers were dried at room temperature for 5 day. The dried red peppers were ground into powder for 2 min using a mixer. The resulting powder was passed through a 250 μm mesh sieve to obtain a homogeneous red powder. The powdered red pepper was stored at − 20 °C until used in the feed. The carotenoid content was determined to be 800 mg/kg of red pepper powder. Approximately 100 g of red pepper powder was obtained from 1 kg of fresh pepper. All ingredients were homogeneously mixed in the mixing bowl during diet preparation. Water was added to the diets to achieve a moisture level of 25%. Experimental diets were passed through a meat grinder (1.5 mm). Then, the experimental diets were dried at room temperature and crushed into 1.5 mm pellets. Prior to usage, experiment diets were stored at 4 °C. Crude protein, lipid, fiber, ash, and moisture levels of diet were assessed using standard AOAC^31^.

**Table 1:** The formulation and proximate composition (%) of the experimental diets.

	Control	3%	7%	11%	15%
Soybean meal	20.00	20.00	20.00	20.00	20.00
Fish meal	35.00	35.00	35.00	35.00	35.00
Wheat flour	35.00	32.00	28.00	24.00	20.00
Red pepper	0.00	3.00	7.00	11.00	15.00
Sunflower oil	7.00	7.00	7.00	7.00	7.00
Vitamin ^1^	2.00	2.00	2.00	2.00	2.00
Mineral ^2^	1.00	1.00	1.00	1.00	1.00
*Proximate composition (%)*					
Crude protein	37.13	37.15	37.17	37.19	37.21
Crude fiber	1.81	1.92	2.07	2.22	2.36
Crude fat	10.91	11.12	11.40	11.67	11.95
Crude ash	9.61	9.64	9.68	9.72	9.76
Digestible energy (kcal/kg)	3831	3831	3831	3830	3830

^1^ Vitamin premix contained the following per kilogram; 2400 mg vitamin E, 4,000,000 IU vitamin A, 40,000 mg vitamin C, 4000 mg vitamin B1, 480,000 IU vitamin D3, 2400 mg vitamin K3, 1200 mg folic acid, 6000 mg vitamin B2, 10,000 mg Cal.D, 4000 vitamin B6, 10 mg vitamin B 12,60,000 mg inositol, 100 mg D-Biotin.

^2^ Mineral premix contained the following per kilogram; 75,000 mg zinc, 23,750 mg manganese, cobalt 2000 mg, copper 5000 mg, selenium 100 mg, iodine 2750 mg, magnesium 200,000 mg.

### Experimental conditions

Juvenile jewel cichlid (*Hemichromis guttatus*) were obtained from aquaculture laboratory of the Egirdir Fisheries Faculty, Isparta University of Applied Science, Türkiye. Before the experiment began, fish (3.43 ± 0.03 g) were adapted to the experimental environment for two weeks. The experiment was carried out in fifteen aquaria (70 × 30 × 40 cm). Fifteen fish were randomly placed into each aquarium, with three replicates for each treatment. The fish were fed ad libitum twice daily for a period of 90 days. Feces and feed waste were siphoned from a quarter of the aquarium water every day. The average oxygen, temperature, and pH content of the freshwater utilized in the experiment were 6.07 ± 0.13 mg L^-1^, 25.3 ± 2.03 °C and 7.4 ± 0.20, respectively. The study was approved by Animal Experimental Ethics committee with reference number 2018 HADYEK-02.

### Histological examination

Nine fish (3 per replicate) were randomly selected from each treatment, euthanized with an overdose of clove oil (180 ppm)^32,33^, and their gonads were collected. Samples were fixed in 10% neutral buffered formalin. After routine processing of the formalin-fixed samples using automatic tissue processing equipment (Leica ASP300S; Leica Microsystem, Nussloch, Germany), the samples were embedded in paraffin, and 3 μm sections were prepared using a Leica RM 2155 rotary microtome (Leica Microsystem, Nussloch, Germany). Sections were stained with eosin and hematoxylin and examined using a light microscope. Oocyte counting was performed to evaluate ovarian status. Oocyte numbers were calculated based on stages (I-V). To achieve this, five randomly selected regions from each ovary were examined under a X40 objective lens. The evaluation included the identification of oocyte stages as described in previous studies^34,35^. Oocyte diameters and the thickness of the zona radiata were measured in randomly selected regions of at least 10 oocytes in the same developmental stages. The oocyte counts were performed in 5 distinct fields on each slide for each fish. Image analysis was conducted using the ImageJ program (National Institutes of Health, Bethesda, MD, version 1.48). Microphotographs were captured using the Database Manual Cell Sens Life Science Imaging Software System (Olympus Co., Tokyo, Japan)^35^.

### Antioxidant activity

Five fish from each experimental group were randomly chosen and anesthetized with 180 ppm clove oil. For antioxidant analysis, muscle tissue from each fish was homogenized in 1:9 (w/v) phosphate buffered saline solution (pH: 7.4) first in a homogenizer (IKA Ultra-Turrax T25 Basic; Labortechnic, Staufen, Germany), and then with a sonicator (UW-2070 Bandelin Electronic, Germany). Following homogenization, samples were centrifuged at 10.000 rpm for 10 min and SOD and CAT analyses were performed on the supernatant of each fish.

SOD activity was measured spectrophotometrically using a Randox commercial kit attached to an Olympus AU 2700 (Japan) autoanalyzer. The principle of the method is based on the formation of uric acid and O_2_^−^ radical from xanthine because of the reaction catalyzed by xanthine oxidase and the subsequent reaction of the O_2_^−^ radical with INT (2-(4-iodophenyl)-3-(4-nitrophenol)-5-phenil tetrazolium chloride) to form a red colored compound form a zone. SOD activity is assessed by the degree of inhibition of this reaction. SOD activity values of the tissue are expressed as U/mg protein^36,37^.

CAT activity was studied according to the Aebi (1984) method^38^. The method is based on the principle of spectrophotometric measurement of the absorbance of H_2_O_2_ 240 nm during the conversion of H_2_O_2_ to water and molecular oxygen in the presence of CAT. First, the first reagent (pH 7.0 50mM phosphate buffer) was prepared. Then, the second reagent was prepared by mixing the first reagent and 30% H_2_O_2_. Tissue sample, first reagent and second reagent were mixed and absorbance values at 0 s and 30 s were analyzed. The results expressed as qu/mg protein.

### Determination of LD50 dose

A. hydrophila A210^39^ was grown in 10 ml of TSB at 25 °C for 24 h and harvested with centrifugation at 5000 g for 15 min at 4 °C. The pellet was resuspended in PBS (pH 7.0). Duplicate groups of 10 fish were infected with A. hydrophila at doses 105, 106 and 107 cfu ml − 1 by immersion bath at 30 min^40^. Mortalities were recorded daily for 15 days and LD50 value was calculated as 106 cfu ml^− 1^.

### Experimental infection

After 90 days of the feeding experiment, all groups were challenged with *A.hydrophila* 210. Ten fish were stocked in separate aquaria (70 × 30 × 40 cm) as 2 replicates. Fish were challenged with *A. hydrophila* 210 at LD_50_ dose (10^6^ cfu ml ^− 1^) by immersion bath^40^. Mortalities were recorded daily for 15 days. *A. hydrophila* 210 was reisolated from the internal organs of freshly dead fish after the mortalities.

### Statistical analysis

SPSS 15.0 software was used to compare oocyte counting and antioxidant levels between groups. Initially, the data were analyzed for normality of distribution using the Shapiro-Wilk test. Since the data showed a normal distribution (*P* > 0.05), comparisons between the groups were made using one-way analysis of variance (ANOVA). Duncan’s multiple range test was used to determine the significant variation. The variables are presented as means ± standard deviations. Values of *P* < 0.05 considered statistically significant.

## Results

### Oocyst stages

**Fig. 1:**
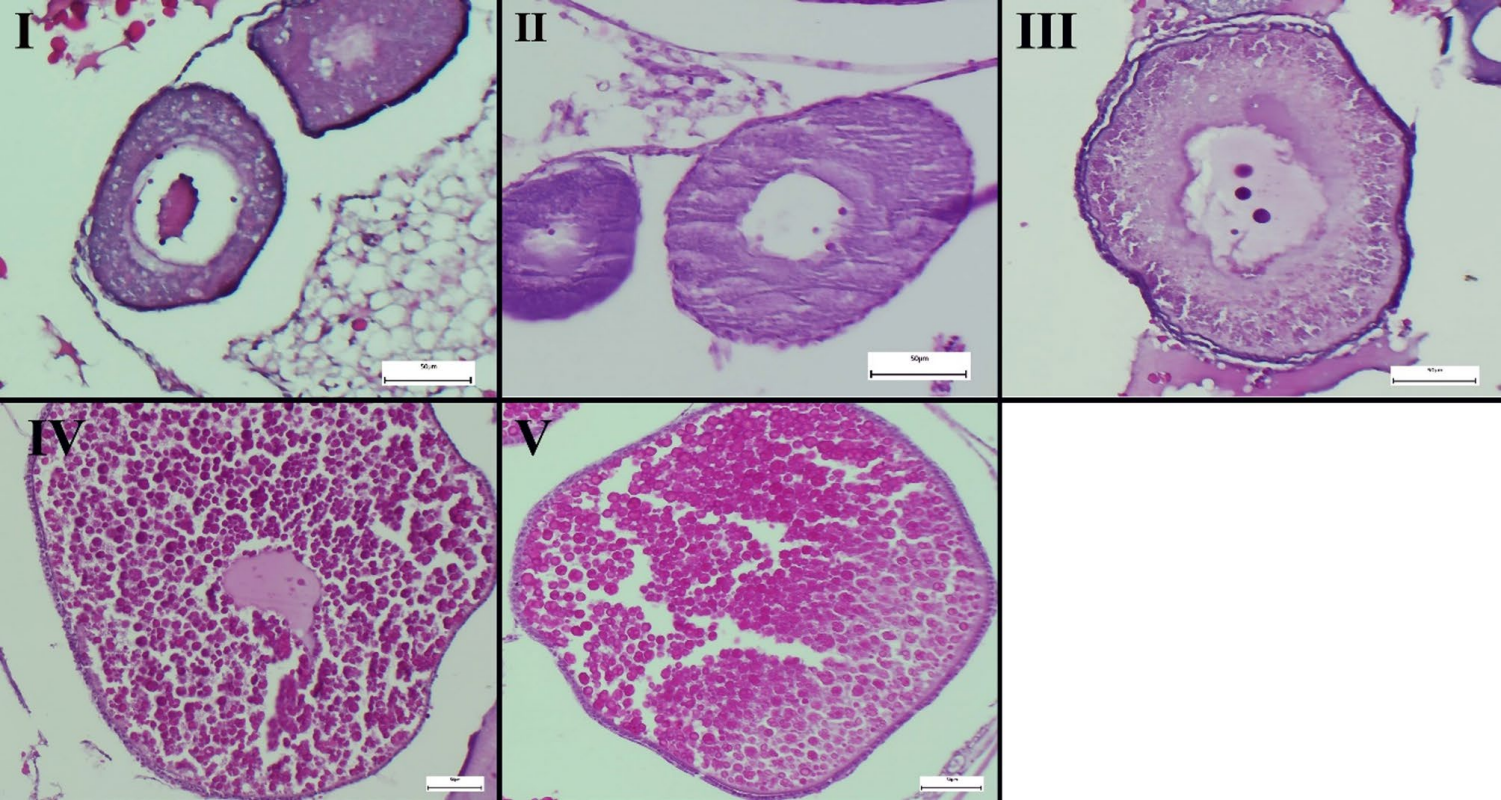
Representative histological section demonstrating the sequential stages of oocyte maturation (Stages I–V), H&E staining, scale bar = 50 μm.

The assessment of oocyte stages was determined according to the criteria in Table 2. Oocytes in stage I (pre-vitellogenic) ranged in size from 55 to 68 μm (61.00 ± 6.55) and had an irregular shape without a zona radiata. Oocytes in stage II (pre-vitellogenic) were ellipsoid in shape, 155 to 187 μm (172.33 ± 16.16) in diameter along the long axis, and had granular cytoplasm. The typical wavy margins and large cortical vacuoles of stage III (pre-vitellogenic )oocytes were present, and their diameter ranged from 410 to 432 μm (420.66 ± 11.01). The zona radiata on these oocytes measured 2 to 4 μm in thickness. Oocytes in stage IV (vitellogenic) had a diameter of 762 to 833 μm (798.33 ± 35.52), were spherical in shape, and had a zona radiata that was 7 to 10 μm thick. Oocyte diameter in stage V (vitellogenic) ranged from 840 to 885 μm (865.00 ± 22.91) with no nucleus or granules, and zona radiata thickness was thinner than at earlier development stages (Fig. 1).

**Table 2:** Description of oocyte developmental stages of jewel cichlid fed red pepper.

Oocyte stage	Size range (µm)	Shape	Cytoplasm characteristics	Zona radiata thickness
I (Immature)	55–68	Irregular	–	Absent
II	155–187 (long axis)	Ellipsoid	Granular	Absent
III	410–432	Spherical with wavy margins	Large cortical vacuoles	2–4 mm
IV	762–833	Spherical	–	7–10 mm
V (Mature)	840–885	Spherical	Absent (no nucleus and granules)	Thinner than earlier stages

### The number of oocytes

**Fig. 2:**
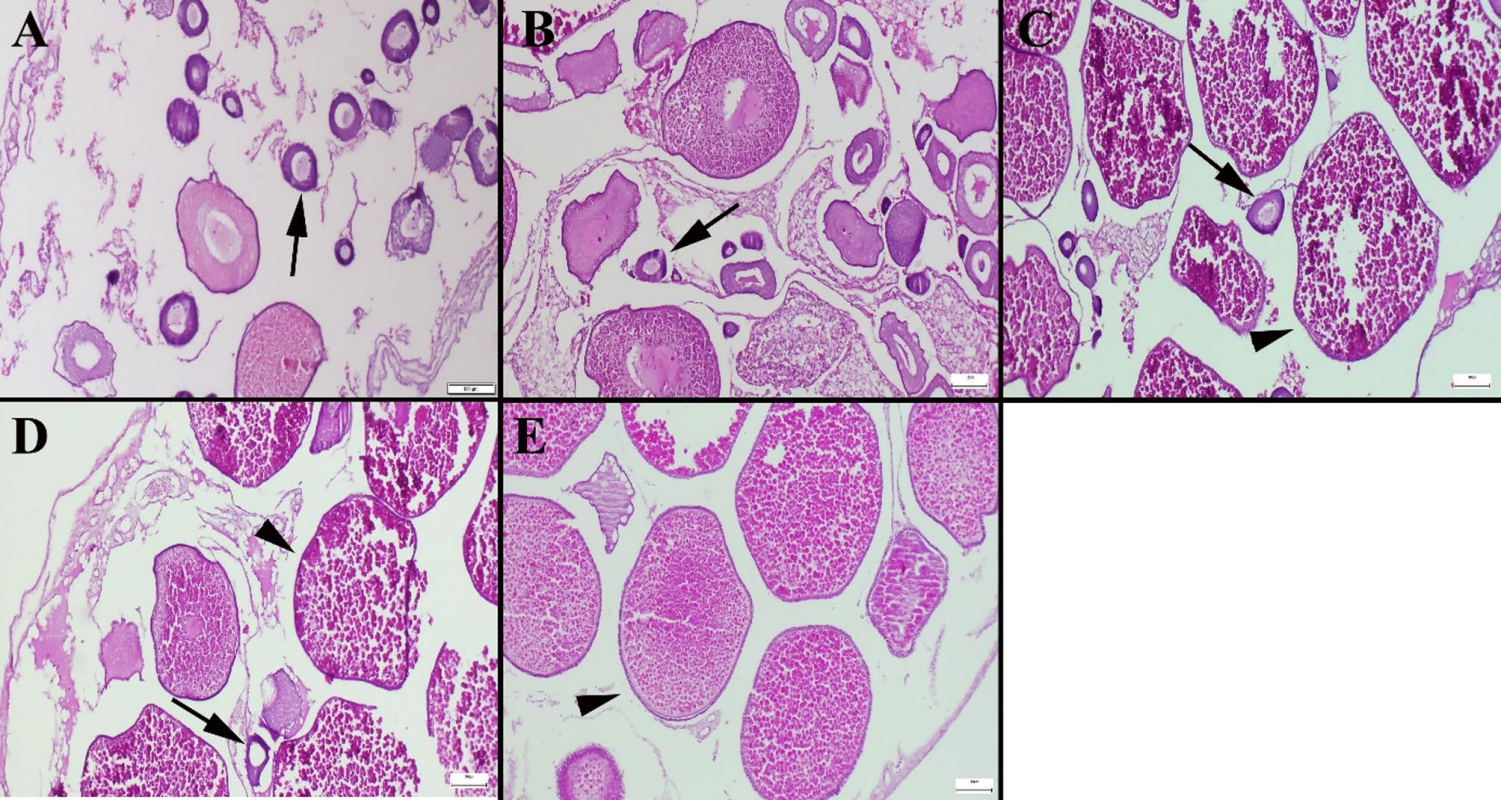
Microscopical appearance of ovarian tissue across experimental groups. (**A**) Ovarian sections from the control group predominantly showed oocytes at immature stages. (**B**) The 3% pepper group exhibited a similar distribution of immature oocytes as seen in the control group. (**C**) In the 7% pepper group, an increase in mature oocytes accompanied by a reduction in immature forms was observed. (**D**) The 11% pepper group showed a further increase in mature oocyte count. (**E**) Notably, the 15% pepper group exhibited a marked increase in mature oocytes, indicating prominent follicular maturation. Arrows indicate immature oocytes, and arrowheads indicate mature oocyte stages. H&E staining, scale bar = 100 μm.

A large number of normal vitellogenic oocytes at various stages were observed in the control group. Significant increases in the number of mature oocytes were observed in the groups treated with pepper supplementation. The inclusion of 11% and 15% red pepper in the diets resulted in an increase in the number of mature oocytes (Table 3; Fig. 2).

**Table 3:** Oocyte counts in each stage of fish fed with red pepper.

Groups	Stage				
	I	II	III	IV	V
Control	24.00 ± 1.41^a^	22.50 ± 3.53^a^	22.50 ± 3.53^ab^	21.00 ± 5.65^as^	10.00 ± 7.07^a^
3%	27.50 ± 3.53^a^	22.50 ± 3.53^a^	22.50 ± 3.53^ab^	15.00 ± 7.07^a^	12.50 ± 3.53^a^
7%	21.50 ± 2.12^a^	20.50 ± 7.77^ab^	29.00 ± 1.41^b^	14.00 ± 1.41^a^	15.00 ± 7.07^a^
11%	8.50 ± 4.94^b^	10.00 ± 0.00^b^	17.50 ± 3.53^a^	31.50 ± 2.12^b^	32.50 ± 3.53^b^
15%	8.50 ± 2.12^b^	12.50 ± 0.70^ab^	17.00 ± 4.24^a^	32.00 ± 2.82^b^	30.00 ± 0.00^b^

The difference between the means of groups showing different letters in the same column is statistically significant (*p* < 0.001). Data given as mean ± standard deviation (SD).

### Challenge with *Aeromonas hydrophila*

**Fig. 3:**
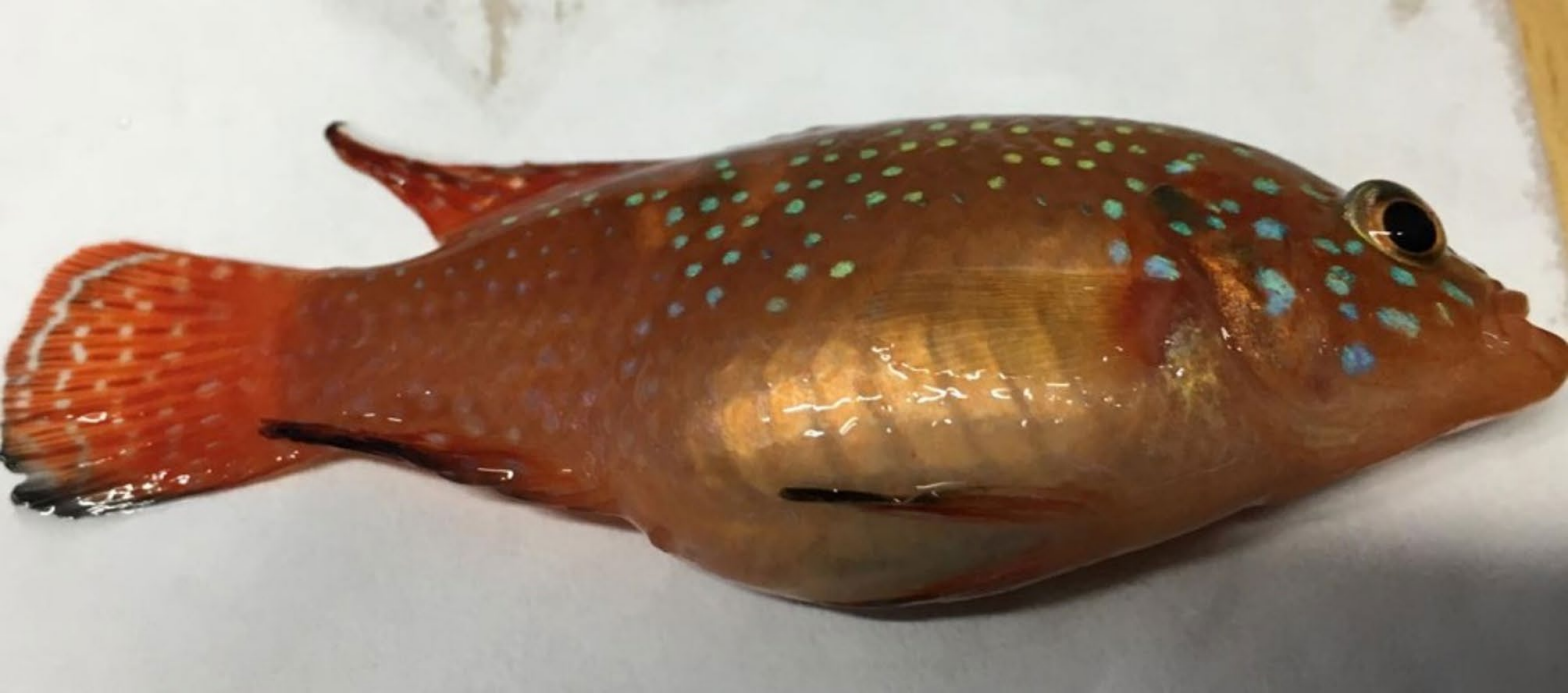
Unilateral exophthalmos accompanied by abdominal swelling resulting from the accumulation of ascitic fluid.

**Fig. 4:**
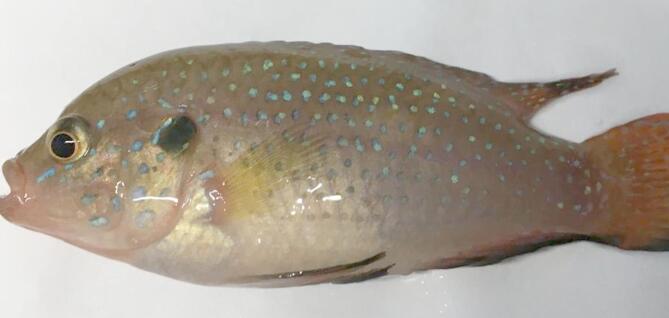
Darkening and hemorrhagic lesions in the jaw, mouth, eye, and pectoral fin base.

Red pepper enhanced disease resistance against *A. hydrophila* 210. As the level of red pepper in the feed increased, protection against *A. hydrophila* infection decreased. Mortality rates in 3% red pepper (20.06 ± 0.00), 7% red pepper (20.06 ± 0.00 ), and 11% red pepper (34.93 ± 6.80) groups were significantly lower than in the control group (50 ± 0.00) and 15% red pepper (50 ± 0.00) (*P* < 0.05). The best protection against *A. hydrophila* 210 was found in the group fed with 3% and 7% red pepper feed (Table 4). Infected fish exhibited dark coloration, monolateral exophthalmos, degeneration in all fin, abdominal swelling due to ascitic fluid accumulation, pale of liver, hemorrhage in the jaw, mouth, eye and base of pectoral fin (Figs. 3 and 4).

**Table 4:** Cumulative mortality (%) in fish challenged with *A. hydrophila*.

Groups	Cumulative mortality (%)
3%	20.06 ± 0.00^a^
7%	20.06 ± 0.00 ^a^
11%	34.93 ± 6.80^b^
15%	50.00 ± 0.00 ^c^
Control	50.00 ± 0.00 ^c^

Data shown with different letters at same column are indicate significant differences *p* < 0.05). Data given as mean ± standard deviation (SD).

### Antioxidant activity

SOD activity increased with the increase in the level of red pepper in the feed. The highest SOD activity was found in fish fed with 11% (206.31 ± 5.88) and 15% (205.39 ± 2.39) red pepper supplementation diets (*P* < 0.05). SOD activity in groups of fish fed with other levels of red peppers was found to be similar to the control (*P* > 0.05). Catalase (CAT) activity relatively increased in fish fed with 7% (3.65 ± 3.00) and 11% (5.03 ± 0.39) red pepper (*P* > 0.05) (Table 5).

**Table 5:** Antioxidant activity in muscle of fish fed with red pepper.

Experimental groups	SOD (U mg protein^− 1^)	CAT (qu mg protein^− 1^)
Control	47.52 ± 1.06^a^	0.51 ± 0.45
3%	47.35 ± 0.06^a^	0.18 ± 0.18
7%	45.54 ± 1.01^a^	3.65 ± 3.00
11%	206.31 ± 5.88^b^	5.03 ± 0.39
15%	205.39 ± 2.39^b^	2.04 ± 1.00

Data shown with different letters at same column are indicate significant differences (*P* < 0.05). Data given as mean ± standard deviation (SD).

## Discussion

Medicinal plants are used in the prevention and control of diseases because they stimulate the immune system of fish^41^. In this context, red pepper, which is rich in phenolic compounds such as flavonoids and phenolic acids, contributes to the prevention of diseases^16^. These compounds may help improve fish health by reducing oxidative stress, enhancing immune responses, and inhibiting pathogenic microorganisms. In this study, using 3–11% red pepper in the jewel cichlid diet provided disease resistance against *A. hydrophila*. The best protection was found in the group fed with 3% and 7% red pepper diet. This result can be explained that the phenolic compounds found in red pepper may provide protection against diseases by acting as antioxidants and antimicrobials^12,13,15^. Similarly, Firouzbakhsh et al.^15^ also informed that red pepper extract could enhance immune responses against to *Yersinia ruckeri* in rainbow trout. However, 15% red pepper did not protect jewel cichlids in this study. Similarly, Yiğit et al.^29^ informed that 15% red pepper supplementation to yellow tail cichlid diet not protected against *A. sobria* infection. It is thought that this result may be due to the suppression of the immune system in jewel cichlids by feeding high amounts of red pepper.

Antioxidant enzyme activity is a potential indicator of oxidative stress, the extent of cell membrane and DNA damage, and the elimination of excess ROS and H_2_O_2_^42,43^. A strong antioxidant defense, provided by sufficient SOD activity, helps to manage this oxidative stress, preventing excessive damage to the tissues and supporting the functions of immune cells^44^. In this study, supplementation with red pepper (especially 11% and 15%) significantly increased the enzymatic activities of SOD in rainbow trout. These results suggest that red pepper can defend against pathogens by modulating host immunity and alleviating pathogen-induced oxidative stress. However, it was found that it provided disease resistance against *Aeromonas hydrophila* when used at 11% doses, but did not provide disease resistance when used at 15%. The possible reason for this may be that the use of 15% red pepper suppresses the immune system. In a study conducted with red pepper, SOD activity did not change in the liver of olive flounder (*Paralichthys olivaceus*) fed with paprika extract^45^. Differences in results may be due to differences in fish species.

Reproductive performance of fish is strongly influenced by the nutritional status^46–48^. Aquatic animals accumulate carotenoids in their gonads. Carotenoids are thought to be essential for aquatic animal reproduction. Addition of carotenoids to salmon and red sea bream diet increased ovary development, fertilization and hatching^49^. There is no information on effects of red pepper supplementation on ovary development in cichlid. In the present study, the number of mature oocytes increased with the addition of red pepper at 11 and 15% to diets of jewel cichlid. Similarly, Watanabe & Vassallo Agius^20^ reported that paprika powder supplementation improved egg production and egg quality of yellowtail (*Seriola quinqueradiata*). Vassallo Agius et al.^50^ reported that number of fertilized eggs increased with addition of paprika powder to yellowtail diet. Scabini et al.^21^ also informed that carotenoids supplementation to diet improved reproductive performance in gilthead seabream (*Sparus aurata*). Adding astaxanthin (a type of carotenoid) to the diets of broodstock has been shown to enhance egg quality in several species, including yellowtail^51^, striped jack (*Pseudocaranx dentex*)^52^, and sea urchin (*Lytechinus variegatus*)^53^. Additionally, Agius et al.^30^ observed that the egg diameter of yellowtail fish fed diets supplemented red pepper increased by the 16th week. Similar results were also found in this study.

## Conclusions

In conclusion, the addition of red pepper at 11 and 15% to jewel cichlid diet increased antioxidant enzyme activity (SOD) in the muscle. In addition, the number of mature oocytes increased with the addition of red pepper at same doses to diet. Moreover, 3–11% red pepper provided disease resistance to A. *hydrophila* infection. Therefore, using 11% red pepper can be safely used to enhance fertility and antioxidants status, and provide diseases resistance against *A*. *hydrophila* infections in jewel cichlid.

## Data Availability

Sequence data that support the findings of this study have been deposited in the GenBank with accession number (A. hydrophila A210) MGO62886.
